# Emergent Group Level Navigation: An Agent-Based Evaluation of Movement Patterns in a Folivorous Primate

**DOI:** 10.1371/journal.pone.0078264

**Published:** 2013-10-21

**Authors:** Tyler R. Bonnell, Marco Campennì, Colin A. Chapman, Jan F. Gogarten, Rafael A. Reyna-Hurtado, Julie A. Teichroeb, Michael D. Wasserman, Raja Sengupta

**Affiliations:** 1 Deptartment of Geography, McGill University, Montreal, Quebec, Canada; 2 Max Planck Institute for Evolutionary Anthropology, Leipzig, Germany; 3 Deptartment of Anthropology & McGill School of Environment, McGill University, Montreal, Quebec and Wildlife Conservation Society, Bronx, New York, United States of America; 4 Deptartment of Biology, McGill University, Montreal, Quebec, Canada; 5 ECOSUR-Campeche, Lerma, Cempeche, Mexico; 6 University of California Santa Cruz, Anthropology Department, Santa Cruz, California, United States of America; 7 Deptartment of Anthropology, McGill University, Montreal, Quebec, Canada; University Toulouse 1 Capitole, France

## Abstract

The foraging activity of many organisms reveal strategic movement patterns, showing efficient use of spatially distributed resources. The underlying mechanisms behind these movement patterns, such as the use of spatial memory, are topics of considerable debate. To augment existing evidence of spatial memory use in primates, we generated movement patterns from simulated primate agents with simple sensory and behavioral capabilities. We developed agents representing various hypotheses of memory use, and compared the movement patterns of simulated groups to those of an observed group of red colobus monkeys (*Procolobus rufomitratus*), testing for: the effects of memory type (Euclidian or landmark based), amount of memory retention, and the effects of social rules in making foraging choices at the scale of the group (independent or leader led). Our results indicate that red colobus movement patterns fit best with simulated groups that have landmark based memory and a follow the leader foraging strategy. Comparisons between simulated agents revealed that social rules had the greatest impact on a group’s step length, whereas the type of memory had the highest impact on a group’s path tortuosity and cohesion. Using simulation studies as experimental trials to test theories of spatial memory use allows the development of insight into the behavioral mechanisms behind animal movement, developing case-specific results, as well as general results informing how changes to perception and behavior influence movement patterns.

## Introduction

Many species face the need to find resources that vary both spatially and temporally, and observed patterns of movement often suggest complex behavior to face these challenges. The mechanisms and processes driving these movement patterns have been a topic of considerable debate, suggesting mechanisms such as spatial memory, internal time measures, communication, and reliance on conspecifics [1-3]. These types of questions can, and are being, examined with many different approaches, and largely focus on defining how, why, and where individuals move [4]. Simulation models, which attempt to represent individuals, show a promising multidisciplinary approach which can incorporate developments in biology, cognitive science, and ecology in testing movement hypotheses. In representing individuals, biological understanding can inform what sensory and movement capabilities individuals are capable of, whereas developments in cognitive science inform how individuals process this incoming information, and how they choose actions in response to this information (e.g., rule-of-thumb, re-enforced learning) [5,6]. The inclusion of an ecological understanding further recreates the conditions under which individual movement and foraging occurs (e.g., social hierarchies, predation risk). Agent-based modeling has been used to model movement patterns [7,8], and facilitates multi-disciplinary approaches [9]. The agent-based modeling approach has shown success as an experimental tactic, in which competing models of micro-level description are compared against emergent macro-level patterns [10,11]. Using this experimental approach and focusing on defining individual perceptions and behaviors, it is possible to effectively test various individual movement hypotheses against real-world observed group movement data.

Primates are good candidates to explore this approach, and test how it can be used in developing and refining behavioral theories of movement. Primates have been the target of numerous field studies, which have convincingly demonstrated that they are capable of remembering food locations [12-15]. Various hypotheses have been proposed regarding how primates retain and use information about their landscapes, including the use of Euclidean, landmark, or route based cognitive maps [12,15]. The Euclidean memory hypothesis involves organisms retaining/memorizing the locations of resources so they can calculate distances and angles irrespective of their current surroundings [16,17], whereas both route and landmark based mechanisms are topological, relying on site association. In this case individuals calculate distance and angles to a subset of remembered sites associated with a familiar site on the landscape (i.e., I am at site A and I know site B is nearby). Both hypotheses have received support [17-19]; however, testing between them and determining what an animal remembers or how it uses this memory has proven extremely difficult in a field setting [15]. The difficulty of testing such hypotheses in the field has encouraged the development of computer generated models of foraging behavior [20-25]. 

 To add to existing experimental data, we built simulated primates that are able to perform some actions of a real primate (e.g., move, eat, digest) and sense important characteristics of their surroundings (e.g., food, predation risk, landscape structure, group mates, non-group mates). Once we had defined a primate’s capabilities and sensory inputs, we then explored decisions at the individual level. By defining individual level behavior and examining group level movement we were able to validate and compare between behavior models using different assumptions.

**Figure 1:**
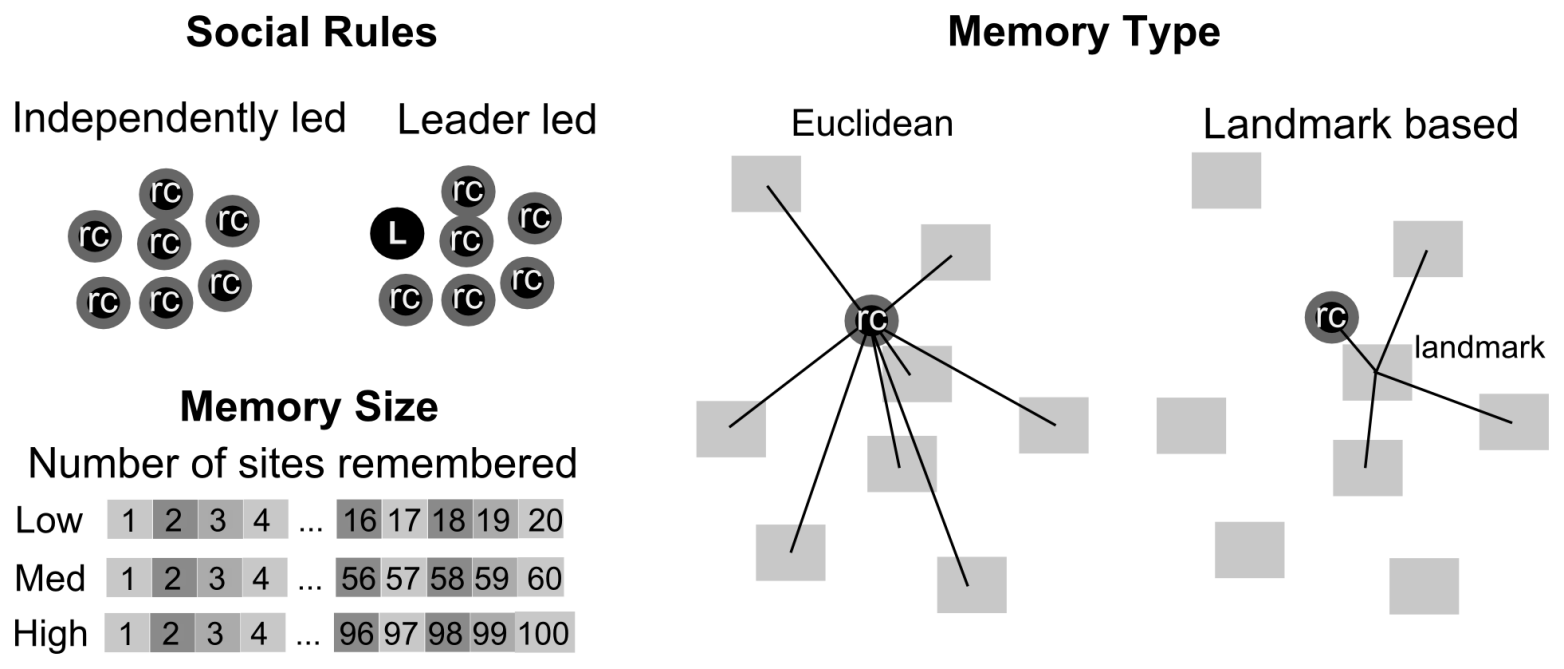
Simulated primate agents (dots), each representing a foraging hypothesis examining the effects of group social rules, memory type, and amount of memory on foraging behavior.

We apply this approach to the red colobus monkey (*Procolobus rufomitratus*) where we examined simulated agents (12 types), representing alternative hypotheses, each with varying social interactions, abilities to retain spatial information, and different methods of using this spatial memory (Figure 1). We ran simulated foraging trials of these different primate agents, and described the resulting group level foraging patterns. Comparing the simulated group level patterns to movement field data, we made inferences about what abilities or attributes are most important in reproducing the observed movement patterns. 

## Methods

### Movement Data

Long-term data on red colobus movement were collected from August 2006 to June 2010 in Kibale National Park, Uganda. A single study group, that grew from 59 to104 animals, was followed, and movements recorded on detailed (50 * 50 m grid) trail maps. The data set included 363 follows, with an average length of 7 h per follow, for 2,564 h of observation. Between August 2007 to June 2008, GPS points of the center of the group were recorded in addition to map locations and the group was generally followed from 8:00 until 13:00, five days per week, for 1,388 h of observation. Permission to conduct the research in Uganda was given by the National Council for Science and Technology, the Uganda Wildlife Authority, and McGill University Animal Care.

### Movement model

Model description follows the ODD (overview, design, details) protocol [26], designed to standardize descriptions of individual- or agent-based models. The model was constructed in Java using Repast Simphony, and is available from: (code.google.com/p/emergent-group-navigation/)

### Overview

#### Purpose

 The purpose of the model was to simulate group movement patterns based on various hypotheses of perception and behavioral responses of individual red colobus monkeys. 

#### Entities, State Variables, and Scales

The model was composed of two types of agents: resources and primates. The model’s base was a landscape where resource polygons made up a gridded surface covering an area of 108ha. A scale of 30×30 m was chosen for each polygon, as this roughly estimates the average canopy size of the major trees in which a large proportion of primate social groups would feed [27], thus we assume foraging decisions were made at this scale. Each polygon contained state variables, current amount of resources and maximum resource levels. A resource polygon, if reduced by foraging, was able to re-grow at a constant rate (“grow back rate”), until it reached its maximum resource level. 

 The primate agents foraging on the resource surface are represented as a point in a continuous space, moving among resource polygons. Agents had two state variables; energy level and the desired number of near-by group mates to be considered safe from predation. Each agent also retained a list of remembered sites (i.e., polygons), containing location, and food estimates.

Foraging simulations were run for 6 months, where one time step in the model represented one half-hour and each day was considered to be 13 h (the active period is from 07:00 to 20:00 [28]). A time step of 30 minutes was chosen because it was the unit of measurement used in our long-term field observations [29]. 

#### Process overview and scheduling

**Figure 2:**
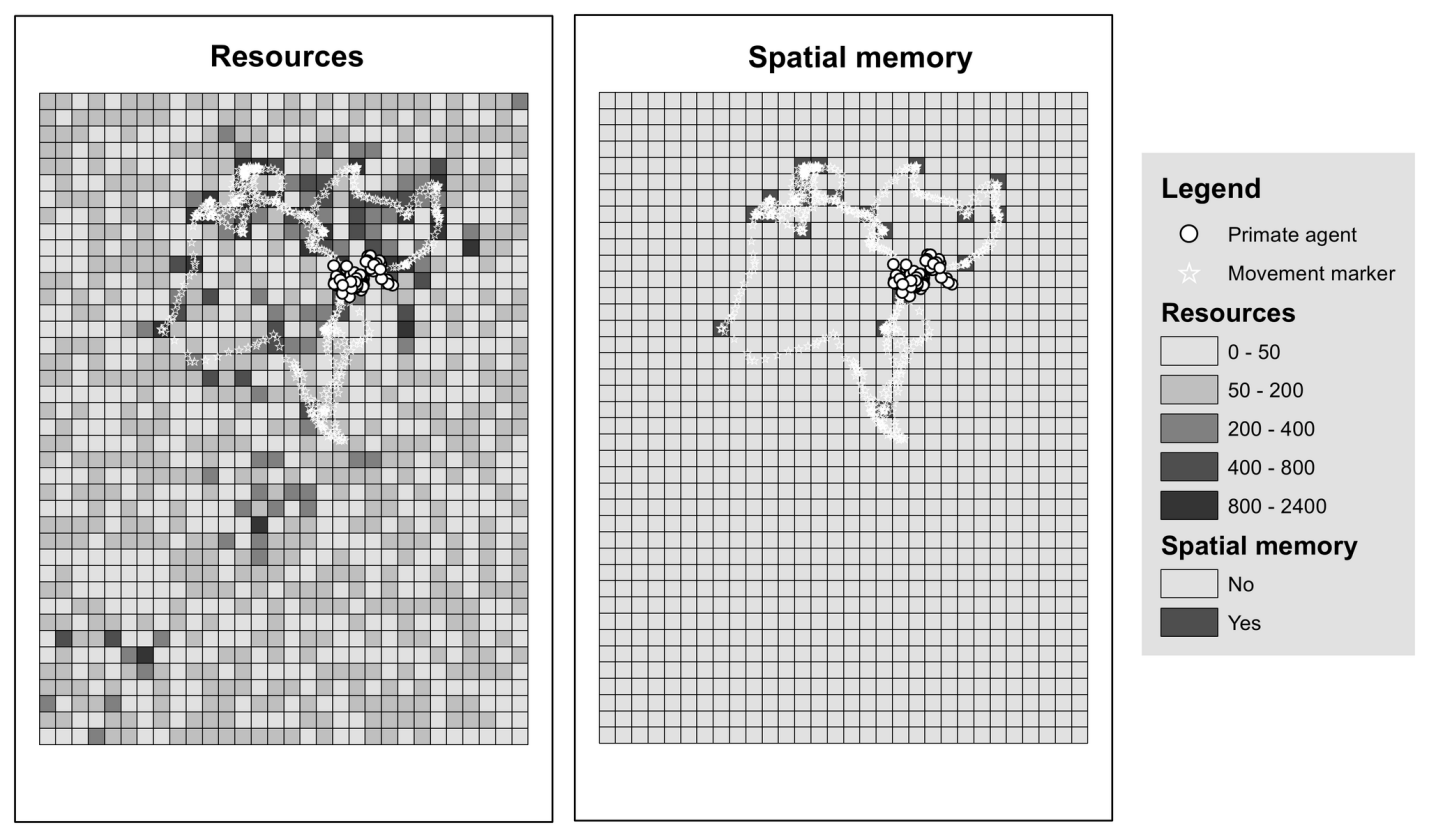
Simulation environment representing: primates (dots), recorded group position (stars), resource and memory landscapes (girds).

Within a time step, each primate agent, the order of which was randomly chosen, responded to internal and external stimulus using a simple behavioral algorithm. The primate agent first made a movement decision: to move towards group mates, a food site, or to simply rest. This movement decision was based on external factors: near-by group mates, visible food sites, as well as internal factors: energy level, desired number of nearby group mates, and remembered food sites (see Submodels: movement-choice, and choose-food-site). Following this movement choice the agent decided whether to try and feed at its given location based on its current energy levels (see Submodels: energetics). Once all primate agents had been processed, the resource polygons performed a re-grow step based on a uniform regrowth parameter, simulating regeneration of food resources (Figure 2). 

### Design Concepts

#### Basic principles

Movement patterns were driven by the interaction between the distribution of resources on a landscape and the primate agent’s capabilities and behaviors. Agents were made to retain varying numbers of resource sites in memory (low-20, medium-60, high-100), and could recall the distance and direction to all these sites (Euclidean) or a subset of these sites based on a nearby landmark (landmark based). Agents also either considered all their neighbours equally (independently led) or depended on a leader (leader led) when considering their safety. This resulted in 12 different types of agents (e.g., an individual with Euclidean memory and low retention, within a group containing a leader) representing different HYPOTHESIS By comparing the movement patterns of groups, each made up of a single type of the 12 possible agents, insight could be gained as to the individual behavioral processes behind observed group level movement patterns for red colobus monkeys. 

#### Emergence

Group moment patterns emerge from individual primate agents balancing their respective safety and feeding requirements. Given different perceptions and behavioral responses, individual agents made different foraging and safety choices altering the movement patterns of the group (S1). 

#### Adaptation

To meet both feeding and safety requirements agents were able to vary the desired number of nearby group mates, allowing them to adjust the level of safety they desired. This allowed the agent, in times of high food stress, to be less attached to the group, prioritizing feeding; whereas, in times of food security the agent could prioritize safety, and reduce predation threats through dilution, although no predation occurred in the model. 

#### Objectives

Primate agents attempted to satisfy, to the best of their ability, their safety and feeding requirements. There was no one optimal solution to this balance, as the optimal solution varied based on the individual, the availability of resources, and the position of group mates at any given time.

#### Sensing

Agents were able to see all resources within visual range (50 m), and detect primate agents within a secondary larger range (200 m). Primate agents were also able to sense their internal energy levels, and to recall sites based on their respective mental representations of the landscape (see Submodels: spatial-memory).

#### Interaction

Primate agents interacted with each other in the model by adjusting their spatial location. By moving towards a desired site, they affected the safety of others within the group. The primate agents also depleted the levels of the resources on the landscape, creating food competition.

#### Stochasticity

At each time step the order that primate agents were processed was randomly chosen. When a primate agent moved to a chosen resource polygon, it was located randomly within its boundaries. Movement at this scale was subject to processes not included in this model (e.g., within group social structure / demographics), and was therefore included as a random factor in the model.

#### Collectives

Primate agents formed a group, where individual interactions affected other group mates foraging decisions. To model within group decision making we included two extreme cases: independently led, where all agents were considered equal, and leader led, where all other agents attempted to stay near a leader for safety (see Submodels: group-foraging). 

#### Observations

At the end of every time step during simulation, the center of the simulated primate group was marked, capturing the position (x,y) and time (step). As groups occasionally performed fission and fusion in the model, a recursive algorithm was implemented to estimate the center of the group. A center point, based on the location of all individuals within the group, was tested to see if a buffer of 100m contained 80% of the group or more. If it did not, the furthest individual from the point was removed and the center was re-calculated on this sub group. This was repeated until the center point passed the 80% rule to ensure that the center point represents that of the majority of the group, and resulted in a center point for each time step.

### Details

#### Input data

To model foraging patterns mimicking observed patterns we developed an estimate of the resource environment in which the observed data was collected. We estimated a resource landscape based on the collected GPS movement data from the red colobus focal group. By overlaying a grid of 30x30 m, covering the home range of the observed group, we counted the number of consecutive hours spent in each grid. Grids with a zero count were weighted by sampling from a uniform random distribution varying from 0 to ¼ h. Each grid (n = 1,200) was thus assigned a relative weight to distribute resources in the simulation. This represents our assumptions of a resource landscape with widely available low quality foods, with specific areas representing high value food sites. This landscape was used in all foraging trails.

#### Initialization

Initialization of the model focused on defining the total amount and the grow-back rate of resources on the simulated landscape. Movement patterns of the simulated group were sensitive to these parameters, and their final values were chosen by fitting simulated group foraging behavior to observed group foraging behavior, specifically, average daily step length, home range size, and group spread. By fitting the model to these movement patterns we, 1) estimated long-term food availability (monthly home range size), 2) estimated short-term food availability (average daily step length), and 3) ensured that individuals were able to meet their energetic and social requirements without breaking group cohesion. Systematic variation of the total amount of resources and grow-back rate was used to select final parameter values (Table 1), which resulted in: an average home range size of all 12 agent types of 20.5 ha (observed: 10-50ha), an average daily step length of 171 m/7h follow (observed: 213 m/7h follow), and an average group spread of 5043 m^2^ (observed: 1878m^2^). The observation data on red colobus was taken from our observed group, as well as published data on neighboring groups [29]. The fit suggested that under the current assumptions, our group model, based solely on the trade-off between safety and food competition, underestimated group movement per day, and overestimated group spread.

**Table 1:** Model parameters describing landscape and primate agents.

**Categories**	**Parameter Name**	**Value**	**Units**
Environment	Resource re-grow rate	8	energy units/step
	Total amount of resources	168000	energy units
Group behavior	Group size	70	individuals
	Safe radius	50	meters
Primate capabilities	Distance to sense food	50	meters
	Distance to sense group mates	200	meters
	Time spent feeding	43	% of day
	Max travel distance (one step)	100	meters
	*Defined by agent type*		
	Foraging decision making	Independent/Leader	social rules
	Memory retention	20,60,100	remembered sites
	Memory Type	Euclidean/Landmark	cognitive map
	Distance of landmark memory	100	meters

 As our simulated foraging trials lasted only 6 months we assumed a relatively static memory model, where individuals were assigned memory at the start of the simulation. Each individual was given memory of the location of top resource sites on the landscape, the number of which was represented by their memory retention abilities. Their memory of how much food at each site was initialized at the start of the simulation, but thereafter was reliant on the last time they were in visual range of the site.

### Submodels

#### Group-foraging

For a social species like the red colobus, group dynamics are important when considering foraging and movement [30,31]. Group-mate choices can influence the behavior of others within the group. Grouping in primates is thought to increase the safety of individuals, by mitigating predation [32], but can have negative effects by increasing food competition [29]. To take this into account we allowed the simulated primate to adapt and balance its needs to gain safety within a group against the increased food competition experienced by being with other foraging conspecifics [29,33]. The animal accomplished this, within our model, by measuring its food intake and in cases where feeding targets were not met, it could lower the number of group-mates needed to meet its safety requirements, thereby prioritizing foraging behavior. In cases where feeding targets were met, it could increase the number of group-mates desired, thereby prioritizing safety. Specifically, at the beginning of its step if the agent had its target energy level it would increase its desired number of nearby neighbours by one, if however the agent was below its target level, and failed to get food during its step, then the agent decreased its desired nearby neighbours by one. This approach mirrored that of [34] and [35] where, allowing individuals to vary their goals between safety and feeding, a group’s behavior showed complex fission-fusion dynamics, such as those seen in the wild. This produced a group that was highly democratic (i.e., independently led), where each individual weighed his/her foraging decisions based on their location, influencing other agents foraging decisions. As an alternative social rule in making group foraging decisions, we added the presence of a leader, where in considering safety all other agents attempt to keep this leader nearby. The leader then played a larger role in making foraging decisions, as its movements influenced others to a greater degree (i.e., leader led).

#### Energetics

Primate agents were driven to forage based on internal energy requirements. Each primate agent aimed to maintain 100 energy units and was considered to be hungry when energy levels fell below this level. The amount eaten per feeding event, was assumed to be constant, and was defined by observed feeding time (red colobus spend 43% of the day feeding [28,29]). Given that the model is divided into 26 half-hour steps, 11 steps (i.e., 43% of their day) should be sufficient to meet their energy requirements. Energy loss per step was constant, and was set as a ratio of target energy divided by the number of steps per day.

#### Movement-choice

Movement choices were made by agents by first comparing their desired level of group safety. If the level of safety was acceptable, agents then made the decision to move towards a desirable food site or to rest based on if they were hungry. If the level of safety was not met, then agents moved towards the average location of the nearest set of group mates, the number of which was defined by the desired number of group mates to be considered safe.

#### Chose-food-site

Food sites, either within visual range or remembered, were compared by ranking them based on the distance and the amount of food expected [20,23],  *I*_(*x*_^′^_,*y*_^′^_)_=*d*((*x*,*y*),(*x* ^′^,*y* ^′^)) / *r*(*x* ^′^,*y* ^′^)where the food site index, I( ), was calculated for a resource at point (*x* ^′^,*y* ^′^) from the primates current location(*x*,*y*), using the Euclidean distance, *d*( ), and the primates estimate of resources*r*( ). Once a food site was chosen, the primate agent would then move towards it. If the food site was contained within the agent’s visual radius and was considered safe, the agent was assumed to move directly to the site. The agent decided if the move was safe based on whether there were enough neighbours nearby at the new site. If the desired food source was beyond this threshold (e.g., from a remembered site) or was not considered safe, then the agent would move to a safe alternative in the direction of the chosen site, moving the agent as far as possible towards their desired site while still remaining safe. By again comparing food sites with the food site index, using the distance to the chosen site as the distance factor, the agent could then choose the best site leading to the desired site.

#### Spatial-memory

Spatial memory was defined in our model as either “Euclidean” or “landmark based.” In the Euclidean framework we assumed agents had a global picture of remembered sites. Under this assumption, primate agents developed a list of all remembered sites, from which they could determine the distance, direction and expected amount of resources. 

 In contrast, using the landmark based memory framework, we assumed agents had knowledge of remembered sites associated with a given landmark. Under this assumption primates could only recall a list of remembered sites associated with the last landmark seen. Landmarks were set as any remembered site, and were associated with any other remembered site within 100m. A primate agent then recalled a list of all sites associated with its current landmark, and could determine the direction, distance and expected amount of resources at these sites. 

 As our foraging trials are short (6 months) we assume a static number of remembered sites, which are not forgotten over time. The amount of resources remembered at these sites however is dynamic. Once the agent had gone out of visual range of a remembered site, it assumed that the resource would grow back at a constant rate each time step, equal to the global environmental “grow back rate.” This assumes that the primate agents within our model have a good idea of the grow-back rate of their resources. When a remembered site comes within visual range again, the agent can update its memory of resource amount. 

### Analysis

The resulting group movement patterns from each agent type were compared against the observed movement patterns. Foraging patterns compared were: monthly spatio-temporal aggregation; daily step-length distribution, and daily path-tortuosity. The distribution of daily step lengths (sum of all group movement in one day), after removing the mean (as this was used in calibrating the model), and the distribution of daily path tortuosity were compared to the observed distribution by using a Kolmogorov-Smirnov test, with the null hypothesis that the observed distribution came from the same distribution as the simulated distribution. The D statistic, measured by the Kolmogorov-Smirnov test, was used as a measure of similarity to the observed distribution and was used to compare between simulation fits to the observed data (e.g., agent type 2 vs. 3). Daily step tortuosity was calculated as a ratio between the total distance traveled and the net distance traveled, log(D_total_ / D_net_^2^). A log transformation was used to normalize the data, whereas the net distance was squared as it commonly increases linearly with step length [36]. Additionally, a measure of skewness for the step length distribution and a t-test for comparing mean path tortuosity were used to compare between observed and predicted movement patterns.

 Estimates of monthly habitat use were made using a spatial-temporal beta-binomial framework (ST-BBD) [37], where time and space in which the animal is moving is subdivided based on a beta-binomial distribution. This approach captures variation in the probability of visiting areas on the landscape; high variability when select few sites are visited often, and low variability when many areas are visited equally. This variation is quantified in this framework by the index of aggregation parameter, θ. A spatial-temporal beta-binomial grid consisting of 30x30 m quadrats, each made up of nine 10x10 m cells recording visits/non-visits during 30 day periods, was overlaid on the simulated and observed movement data. Simulated data was subsampled to meet the collection intensity of the observed data (i.e., 5 days per month and 7 hours per day). 

 To test for the combined effects of group social rules, memory type, and memory retention, a regression tree approach was used. We used the ‘ctree’ function from the ‘party’ package in R (R core development team 2012), a regression tree approach using conditional inference to subdivide the data using a minimum criterion of p<0.05. Step length, tortuosity, and group spread were used as dependant variables in separate regression trees, with group social rule, memory type, and memory retention as explanatory variables.

## Results

### Observed data: red colobus

The red colobus group in Kibale National Park, Uganda had a mean daily step length of 213 m (max 645 m), mean path tortuosity of -1.69, and a mean monthly index of aggregation 0.15 (mean 95% confidence interval: 0.11,0.19) (S2). Daily step length showed a positive skew (0.79), indicating the presence of infrequent long distance travel events. 

### Simulated data compared to observed data

Compared to the observed group, none of the simulated groups were found to have similarly distributed daily step lengths (Table 2). However, an effect of social rule on group movement was apparent, as overall, groups governed by a leader had lower D statistics compared to the independently led groups (Table 2). Leader led groups were also found to have higher positive skew in their distributions, fitting better to the observed positive skew in the observed distribution (Table 2).

**Table 2:** Comparison of daily step length between simulated and observed primate groups; all comparisons with the K-S test were found to be significantly different (p<0.05).

**Kolmogorov-Smirnov - D statistic**				
**Social group**	**Memory**	**20**	**60**	**100**
Democratic	Euclidean	0.32	0.29	0.27
	Topological	0.30	0.27	0.30
Leader	Euclidean	0.25	0.29	0.27
	Topological	0.29	0.22	0.18
**Distribution skew**				
**Social group**	**Memory**	**20**	**60**	**100**
Democratic	Euclidean	-0.08	-0.01	0.04
	Topological	-0.10	<-0.01	0.50
Leader	Euclidean	0.39	0.32	0.32
	Topological	0.94	0.85	0.66

 Comparing simulated and observed distributions of daily path tortuosity suggested that leader led groups with landmark based memory (low-mid memory retention), and leader led groups with Euclidean (mid-memory retention) and landmark based memory (low memory retention) did not differ from the observed distribution (Table 3). Comparing mean levels of daily path tortuosity, groups with landmark based memory and low memory retention in both the leader and independently led groups were not found to differ from the observed mean (Table 3).

**Table 3:** Comparisons of daily step tortuosity between simulated and observed primate groups (mean observed group tortuosity = -1.7); ‘*’ indicates no significant difference at the p=0.05 threshold.

**Kolmogorov-Smirnov test - D statistic**				
**Social group**	**Memory**	**20**	**60**	**100**
Democratic	Euclidean	0.16	0.17	0.19
	Topological	0.10*	0.09*	0.15
Leader	Euclidean	0.14	0.10*	0.17
	Topological	0.10*	0.15	0.13
**Welch’s two-sample t-test**				
**Social group**	**Memory**	**20**	**60**	**100**
Democratic	Euclidean	-3.72	-3.63	-5.05
	Topological	-1.90*	-2.48	-3.01
Leader	Euclidean	-3.08	-2.03	-4.62
	Topological	0.75*	-2.40	-2.67

**Figure 3:**
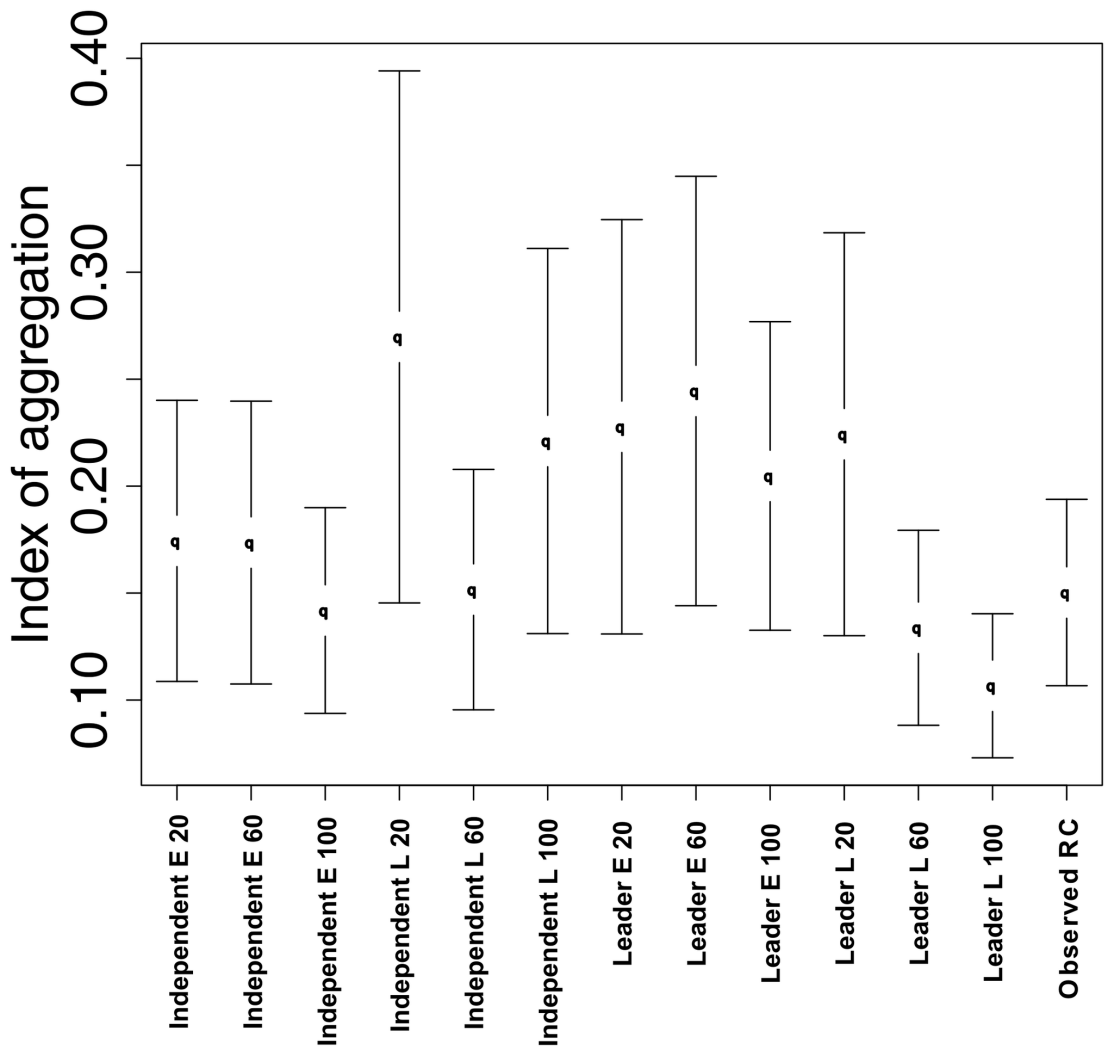
Index of aggregation for the observed and simulated red colobus groups. Error bars on the graph represent 95% confidence intervals.

 Comparing spatial-temporal range use with the observed data revealed that all groups did not differ significantly from the observed red colobus group (95% confidence interval for the difference between the simulation and observed mean). However, there was significant variation between agent types (Figure 3). Groups governed by a leader, with landmark based memory and high memory retention were found to have significantly lower spatio-temporal aggregation than groups governed by a leader and with Euclidean memory type (low, med, and high).

### Effects of social rule, memory type and memory retention

**Figure 4:**
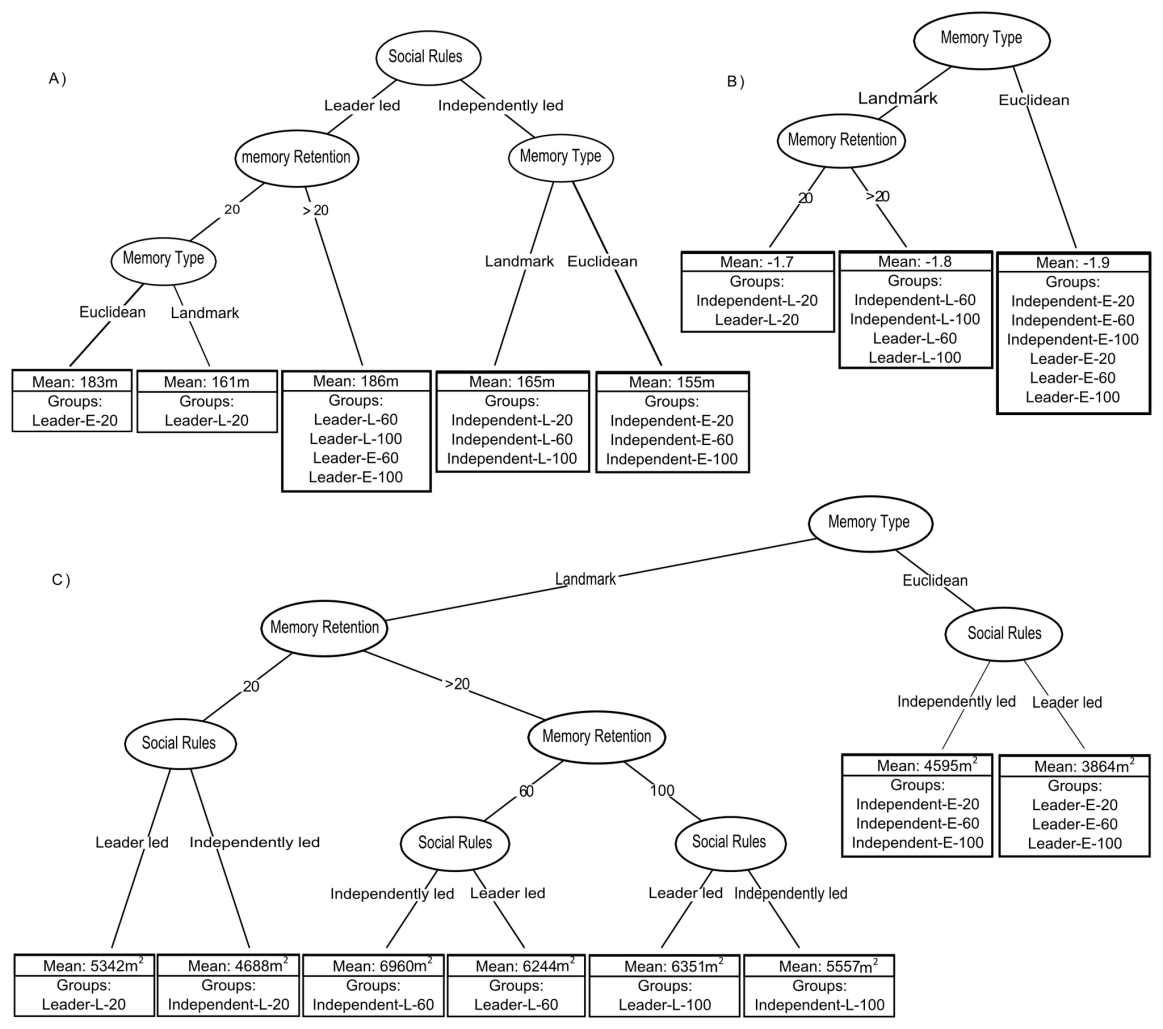
Conditional regression trees of a) daily step length, b) path tortuosity, and c) group spread, using group social rules, memory type, and memory retention as explanatory variables.

Regression tree results revealed a significant effect of social rules, memory type, and memory retention on daily step length (Figure 4). Social rules explained most of the variance in daily step length, splitting the data into leader and independently led group categories. 

Considering daily path tortuosity, the regression tree approach found significant effects of memory type and memory retention, but none for group social rules (Figure 4). Memory type explained the most variance, subdividing groups into Euclidean and landmark based memory types.

Analyses of group spread found that memory type explained most of the variation in group spread (Figure 4). Significantly more heterogeneity in the landmark based memory type was found compared to the Euclidean memory type. In the case of groups with Euclidean memory, significant differentiation was only found with social rules, with no effect of memory retention. The smallest mean group spread was seen in leader led groups with Euclidean memory (3863m^2^), whereas the largest mean group spread was found in the independently led groups with a landmark based memory and high to mid-memory retention (60 sites: 6960m^2^, 100 sites: 6350m^2^).

## Discussion

Using simulated primates, with sensory abilities and simple behavioral responses to these stimuli, we compared simulated movement patterns with the observed movement patterns of a red colobus group. By defining various types of simulated primates, we were also able to test for the general effects of social rules, memory type, and memory retention, giving insight into the processes behind primate movement patterns. 

 Social rules were found to be the most important factor affecting the step length of simulated groups (Figure 4). Leader led groups showed an ability to make longer moves compared to independently led groups which often lacked a clear consensus as to the direction of movement. Furthermore higher levels of discord in the independently led groups masked the effects of individual agent memory retention; whereas, in the case of the leader led groups, memory type and memory retention were found to be significant factors in daily step length (Figure 4). This suggests that social rules governing groups are important factors to consider when elucidating individual foraging strategies/abilities from group level movement patterns. In the case of the leader led groups, when memory retention was high, there was no effect of memory type. This suggests that at high memory retentions the effects of memory type on step length should be small, and that daily step length might not be effective in distinguishing memory use in animals that are thought to have high memory retention.

 In the case of path tortuosity, memory type was the most important factor distinguishing simulated groups. Higher homogeny in groups with Euclidean memory suggests that social rules and memory retention did not affect path tortuosity. In contrast, groups with landmark based memory were found to be significantly affected by memory retention (Figure 4). This is likely due to the fact that as memory retention increased, more nearby food sites were included in memory, allowing high-value sites to be more connected to other high-value sites, creating a network-like structure consisting of high-value sites connected by low-value sites. Group foraging patterns were often found to follow along similar paths or routes, visiting high-value sites and displaying cyclical patterns. This route following behavior was seen in groups with landmark based memory types, as well as groups with Euclidean memory types [21], and resulted in similar measures of home range use between simulated and observed movement patterns (Figure 3).

 Group foraging, as described in our model, resulted in groups that would contract in times of resource abundance and would expand in times of food stress, allowing us to use the measure of group spread as a means of comparing group level foraging competition/success. We found that memory type was the most important factor, followed by memory retention, then finally group social rules in determining group spread. The relative homogeny in the groups with the Euclidean memory type (Figure 4), suggested that group foraging success was not strongly affected by memory retention; that groups with 20 remembered sites did as well as those with 100. In the case of groups with landmark based memory, we saw a division based on memory retention, but not necessarily indicating more foraging success with higher memory retention. We observed that the least successful groups in our model, in terms of group cohesion, were those with landmark based memory and mid/high memory retention. This was partially explained in the model by the fact that as memory retention went up, less valuable sites at the periphery of the home range were included, which, once depleted by a group, were left without a good option to move towards (no, or limited, site specific memories nearby). This resulted in the group relying on visual cues alone to forage, until they found a familiar site. These exploratory events resulted in times of high food competition within the simulated group, resulting in increased group spread.

 Increasing memory retention also had the generally weak effect of lowering the levels of movement aggregation, average θ values being lower for groups with 100 sites compared to 20 sites (comparing groups with similar social rules and memory types), and was most apparent in the leader led group with landmark based memory (Figure 3). These groups likely benefited the most from increases in memory retention as they have limited site specific memories, reducing movement possibilities, and have less group discord compared to the independently led groups. This would allow them to take advantage of the increases in memory retention and use more of the landscape, thereby reducing aggregation in their movement patterns.

 Comparing the 12 types of agents, or hypotheses, to the observed red colobus data, we found that leader led groups fit step length distributions best; whereas, groups with landmark based memory fit distribution of daily path tortuosity best, specifically those with low memory retention (20 sites). Comparing the three groups, which have leader led social rules and landmark based memory with observed movement aggregation revealed that all three are not significantly different than the observed levels. Given these results, the group with leader led social rules regarding foraging choices, and landmark based memory with low (20 sites) memory retention fit best to the observed red colobus data. The suggestion of lower memory retention (i.e., 20 instead of 60, or 100 sites), could possibly indicate that the group is heavily reliant on a smaller subset of food trees, rather than a smaller memory size. As well, when memory was increased in the model, lower resource sites are added resulting in a saturation of benefits from increases in memory retention. With a more detailed description of the dietary requirements of red colobus and a more detailed picture of their resource landscape, the point at which this threshold occurs would likely increase. The selection of the leader led group model was largely due to the lack of a clear consensus as to the direction of movement in the independently led groups. Future work could test the effects of varying within-group decision-making while foraging (e.g., communication, group demographics) to examine its effects on group movement patterns.

## Conclusion

 The approach taken here is based on strong inference, comparing and contrasting which individual level assumptions best fit with observed group level data in an experimental manner. The complex task of representing behavioral responses to stimuli, in a way which represents the behavior of a particular animal, is challenging. Focusing on reproducing group movement patterns, as opposed to those by a single individual, offers a higher level of predictability and is more feasible for collective behavior, rather than individual behavior [38]. As well, work in the field of action selection shows promise in linking developments in cognitive science and behavior [5], and presents a means to expand from the simple behavioral algorithm used in our example, to allow for more individual level behavioral flexibility and complexity seen in many different species [6].

 In a more general context, landscape structure and animal movement patterns can be better mapped due to advances in geographical information systems, remote sensing, and animal tracking (e.g., GPS) technology. This type of approach will be useful to examine such animal movement datasets, and the refinement of such datasets would allow for higher power of falsifiability of various hypotheses, leading to a better behavioral understanding of animal movement.

## Supporting Information

File S1 **Group movement model.** a) Algorithm for basic individual movement behavior, modeling the trade-off between safety benefits of group living against foraging completion costs (see Bonnell et al 2010). The algorithm is run for every individual each time step (start to end blocks). Varying the type and amount of spatial memory affects foraging choices available, whereas social rules affects individual safety requirements. b) Diagram depicting sensory inputs for an individual primate agent (blue circle). Nearby range, defines the area in which group mates add to an individual’s safety, and visual range defines the area in which food sites are visible. Outside of the visible range individuals can remember sites based on the type and amount of spatial memory they possess.
(DOCX)

File S2 **Movement patterns of the observed red colobus group: daily distance traveled, daily path tortuosity, and monthly spatio-temporal aggregation.**
(TIFF)
