# Supplementary material for: Emergent Group Level Navigation: An Agent-Based Evaluation of Movement Patterns in a Folivorous Primate
Source: PLoS One. 2013 Oct 21;8(10):e78264. doi: 10.1371/journal.pone.0078264 (PMC3804626; doi:10.1371/journal.pone.0078264)
Supplement: File S1 — Group movement model. a) Algorithm for basic individual movement behavior, modeling the trade-off between safety benefits of group living against foraging completion costs (see Bonnell et al 2010). The algorithm is run for every individual each time step (start to end blocks). Varying the type and amount of spatial memory affects foraging choices available, whereas social rules affects individual safety requirements. b) Diagram depicting sensory inputs for an individual primate agent (blue circle). Nearby range, defines the area in which group mates add to an individual’s safety, and visual range defines the area in which food sites are visible. Outside of the visible range individuals can remember sites based on the type and amount of spatial memory they possess. (DOCX) [file pone.0078264.s001.docx]

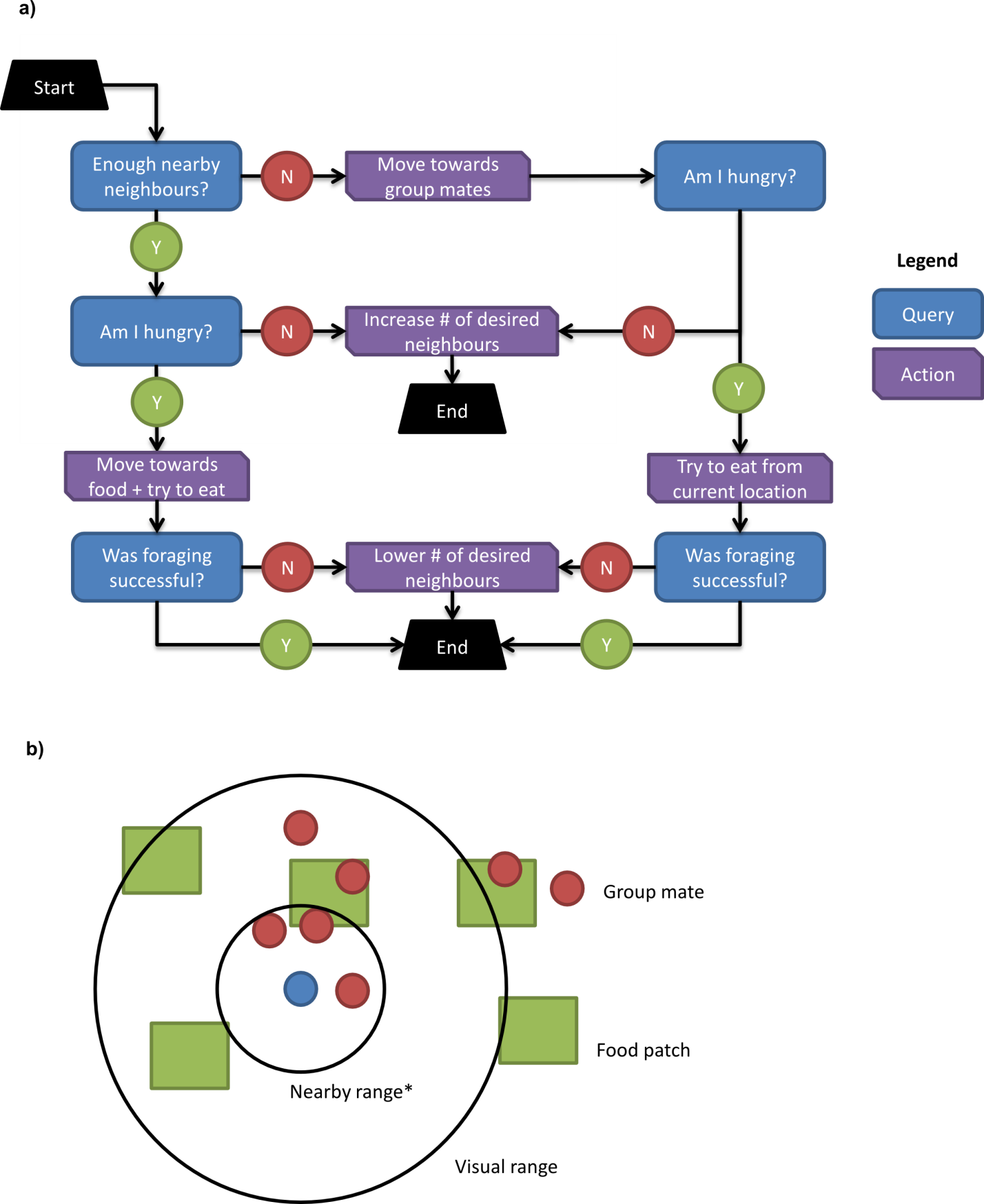


Figure S1: Group movement model. a) Algorithm for basic individual movement behaviour, modeling the trade-off between safety benefits of group living against foraging completion costs (see Bonnell et al 2010). The algorithm is run for every individual each time step (start to end blocks). Varying the type and amount of spatial memory affects foraging choices available, whereas social structure affects individual safety requirements. b) Diagram depicting sensory inputs for an individual primate agent (blue circle). Nearby range, defines the area in which group mates add to an individual’s safety, and visual range defines the area in which food sites are visible. Outside of the visible range individuals can remember sites based on the type and amount of spatial memory they possess.
