# Supplementary figures and images for: Emergent Group Level Navigation: An Agent-Based Evaluation of Movement Patterns in a Folivorous Primate
Source: PLoS One. 2013 Oct 21;8(10):e78264. doi: 10.1371/journal.pone.0078264 (PMC3804626; doi:10.1371/journal.pone.0078264)

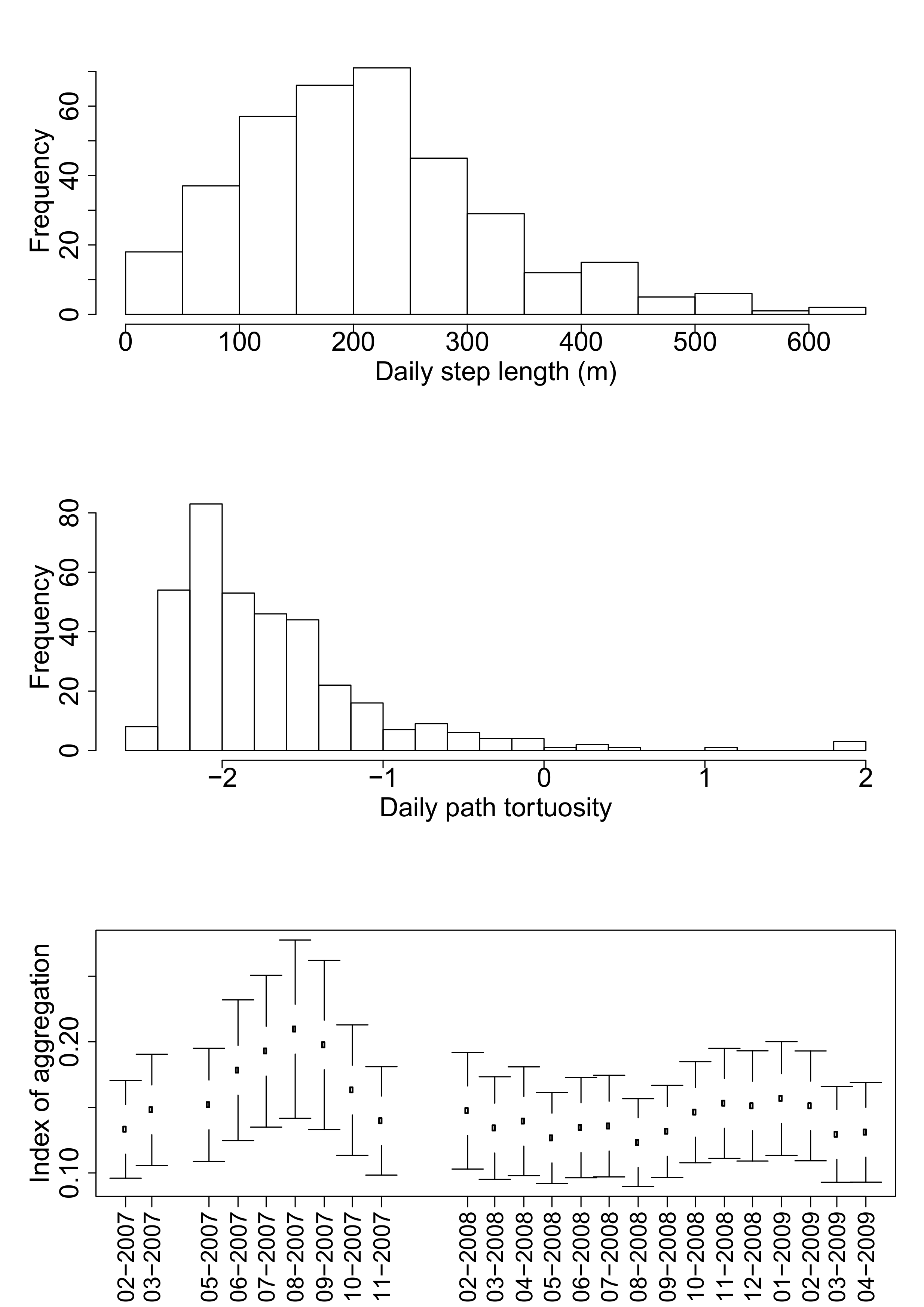

Supplement: File S2 — Movement patterns of the observed red colobus group: daily distance traveled, daily path tortuosity, and monthly spatio-temporal aggregation. (TIFF) [file pone.0078264.s002.tiff]
